# A Narrative Review: The Effect and Importance of Carotenoids on Aging and Aging-Related Diseases

**DOI:** 10.3390/ijms242015199

**Published:** 2023-10-15

**Authors:** Elif Rabia Bakac, Ece Percin, Ayse Gunes-Bayir, Agnes Dadak

**Affiliations:** 1 Department of Nutrition and Dietetics, Faculty of Health Sciences, Bezmialem Vakif University, 34065 Istanbul, Turkey; 2 Institute of Pharmacology and Toxicology, Clinical Pharmacology, University of Veterinary Medicine Vienna, 1210 Vienna, Austria; agnes.dadak@vetmeduni.ac.at

**Keywords:** carotenoids, aging, aging-related diseases, oxidative stress, antioxidant, pro-oxidant

## Abstract

Aging is generally defined as a time-dependent functional decline that affects most living organisms. The positive increase in life expectancy has brought along aging-related diseases. Oxidative stress caused by the imbalance between pro-oxidants and antioxidants can be given as one of the causes of aging. At the same time, the increase in oxidative stress and reactive oxygen species (ROS) is main reason for the increase in aging-related diseases such as cardiovascular, neurodegenerative, liver, skin, and eye diseases and diabetes. Carotenoids, a natural compound, can be used to change the course of aging and aging-related diseases, thanks to their highly effective oxygen-quenching and ROS-scavenging properties. Therefore, in this narrative review, conducted using the PubMed, ScienceDirect, and Google Scholar databases and complying with the Scale for the Assessment of Narrative Review Articles (SANRA) guidelines, the effects of carotenoids on aging and aging-related diseases were analyzed. Carotenoids are fat-soluble, highly unsaturated pigments that occur naturally in plants, fungi, algae, and photosynthetic bacteria. A large number of works have been conducted on carotenoids in relation to aging and aging-related diseases. Animal and human studies have found that carotenoids can significantly reduce obesity and fatty liver, lower blood sugar, and improve liver fibrosis in cirrhosis, as well as reduce the risk of cardiovascular disease and erythema formation, while also lowering glycated hemoglobin and fasting plasma glucose levels. Carotenoid supplementation may be effective in preventing and delaying aging and aging-related diseases, preventing and treating eye fatigue and dry eye disease, and improving macular function. These pigments can be used to stop, delay, or treat aging-related diseases due to their powerful antioxidant, restorative, anti-proliferative, anti-inflammatory, and anti-aging properties. As an increasingly aging population emerges globally, this review could provide an important prospective contribution to public health.

## 1. Introduction

As life expectancy increases, age-related diseases will become more prevalent [1], including biological and cognitive impairments such as mental and emotional deterioration as well as physical weakness [2]. Aging is defined as the progressive impairment of physiological integrity over time and, thus, is a leading risk factor for the development of many diseases including cardiovascular disease, liver disease, neurodegenerative diseases, and diabetes [3].

According to the “Theory of Free Radical Aging”, which was first developed in the 1950s, aging is due to the destruction of biomolecules such as lipids, proteins, and DNA by free radicals resulting from physiological reactions with exogenous and endogenous factors and reactive oxygen [4]. Today, it is fully recognized that reactive oxygen species (ROS) also act as specific signaling molecules that are necessary for maintaining normal cell function. Aerobic metabolism produces continuously reactive forms of oxygen as byproducts, but an increase in their formation under stress can result in severe cellular damage [2,5].

Pro-oxidants contribute to the production of ROS and subsequently the damage of cellular macromolecules, indicating that they increase oxidative stress [5]. In contrast, antioxidants reduce oxidative stress by reducing or eliminating the effects of ROS. Due to an increasing deficiency of endogenous antioxidant mechanisms, elderly individuals are highly vulnerable to oxidative stress [3]. At a certain point, aging processes become inevitable because of the gradual accumulation of oxidative damage caused by the imbalance between pro-oxidants and antioxidants. As these cellular damages gradually progress and accumulate, age-related diseases manifest [4].

Natural compounds with high antioxidant activity have been shown to inhibit aging processes related to oxidative stress [6]. In recent years, carotenoids have garnered considerable attention due to their potent antioxidant, repair, anti-proliferative, anti-inflammatory, and anti-aging properties. Thus, they are considered to be compounds that can help in the prevention of diseases associated with oxidative stress and chronic inflammation [5]. In the present work, research works on the effects of carotenoids on aging and aging-related diseases were reviewed using scientific databases.

## 2. Carotenoids: Properties, Structures, and Functions

Carotenoids are oil-soluble, extremely unsaturated pigments found in plants, mushrooms, algae, and photosynthetic bacteria [7,8]. They are abundant in nature, with over 650 different carotenoids described so far [9]. Because the majority of animals lack the biosynthetic mechanism required to produce carotenoids, they must obtain these natural compounds from fruits and vegetables [10].

Carotene and xanthophyll are the two primary forms of carotenoids. Carotenes comprising β-carotene, α-carotene, and lycopene are non-oxygenated terpenes, while xanthophylls such as lutein, zeaxanthin, astaxanthin, and β-cryptoxanthin contain oxygen in their chemical structures [11]. 

The chemical forms of carotenoids and some sources of carotenoids are given in Table 1. Orange–yellow vegetables and fruits are high in β-carotene, which is a precursor of vitamin A in the human body, and α-carotene. Oranges are rich in α-cryptoxanthin, tomatoes in lycopene, and dark green vegetables in lutein. Egg yolk is a rich source of zeaxanthin and lutein [2,7,12,13,14,15,16,17,18,19,20].

Numerous factors influence the bioavailability of carotenoids, which are advantageous for oxidation due to their unsaturated structure and can be influenced by factors such as light, temperature, and pH [11]. Some prevalent cooking techniques, such as steam cooking and microwave cooking, have little effect on the carotenoid content of nutrients, whereas excessive heat can cause oxidative damage to carotene [2]. 

## 3. Aging

Aging is characterized by the progressive loss of physiological integrity, leading to increased vulnerability to dysfunction and death [1]. However, it is assumed that the signs of aging result from several fundamental molecular changes [3]. The four most fundamental features of these are genomic instability, telomere attrition, epigenetic changes, and loss of proteostasis. In addition, three opposing characteristics are dysregulated nutrient sensing, mitochondrial dysfunction, and cellular aging, while their two integrative features are stem cell depletion and alteration of intercellular communication. 

Oxidative damage provokes fundamental components of pathological pathways thought to lead to signs of aging and many age-related diseases [21]. However, the results of studies testing the oxidative stress hypothesis on this subject are controversial. That is, while observational studies have found detrimental effects of high levels of oxidative stress on disease states, randomized clinical trials examining the effect of antioxidant supplementation on disease states have generally shown null effects.

The decline of senescent cells is partly a result of impaired proteolytic activity. A well-established observation about aging is the tendency for cells to accumulate abnormally or the presence of modified proteins [6]. Additionally, normal cell division is arrested in senescent cells in response to various cellular stresses or DNA damage, accompanied by a pro-inflammatory response, mitochondrial dysfunction, and telomere shortening. 

During aging, individual circumstances may occur such as increased drug use, reduced food consumption due to decreased appetite, or difficulty in absorption [1]. In this case, the functionality and bioavailability of the nutrients received are even more important. Antioxidants are highly important in the prevention of various diseases that may be related to aging as well as in alleviating symptoms and treating the disease [8]. Adhering to a diet with high antioxidant potential has been shown to reduce the risk of mortality from all causes [22]. Researchers have reported that the daily consumption of five servings of fruits and vegetables reduces the risk of death [23].

## 4. The Effect of Carotenoids on Aging

Various mitochondrial defects are thought to contribute to the development of oxidative stress [5]. This situation has been demonstrated by scientific studies to be the main mediator of the aging process and subsequent age-related diseases [5,6]. Therefore, antioxidants targeting the mitochondria are necessary to prevent or slow down these processes and contribute to the health of the cell. It is necessary to examine the interaction of carotenoids, as antioxidant substances, with aging in this context [5,7].

Carotenoids have various functions on human health by showing antioxidant effects. However, carotenoids exert their effects through different mechanisms [7]. Carotenoids are absorbed into the body with many foods consumed as part of a healthy diet [7,9,11]. These accumulate in the skin and protect the skin very effectively against damage caused by UV light, sunburn, and skin aging. Thanks to their chemical structure, containing a large amount of conjugated bonds, carotenoids have a high potential in scavenging ROS such as peroxide radicals or single oxygen molecules [9,10]. The anti-aging potential of carotenoids is mainly based on their promotion of nuclear factor erythroid 2-related factor 2 (Nrf2) migration into the nucleus [10]. After Nrf2 migrates to the nucleus, transcription of antioxidant and detoxifying enzymes begins. Additionally, as a cell divides, its telomeres shorten until it dies, and ROS and inflammation can accelerate telomere shortening [6]. It has been reported that a high dietary β-carotene intake is associated with a longer telomere length [10].

## 5. The Effect of Carotenoids on Aging-Related Diseases

Aging-related diseases such as cardiovascular, neurodegenerative, liver, skin, and eye diseases and diabetes occur due to increasing oxidative stress and ROS [2]. Dietary guidelines recommend consuming fruits and vegetables in appropriate proportions per day to combat the incidence of diseases such as cancer, cardiovascular disease, osteoporosis, and diabetes [24]. Studies that investigated the effect of carotenoids on different organisms with aging-related diseases are presented in Table 2. Dietary carotenoid intake is crucial for protection against diseases as well as for improvement in conditions developed in diseases [7]. In this context, antioxidants such carotenoids are known as very potent ROS scavengers [2] that control autoxidation and prevent an increase in ROS [25].

In addition, the effects of carotenoid types such as lycopene, lutein, β-carotene, astaxanthin, and zeaxanthin on some aging-related diseases are presented in Table 3 and discussed below.

### 5.1. Cardiovascular Diseases and Carotenoids

Cardiovascular diseases are the main cause of high mortality and morbidity in Western countries and are associated with oxidative stress and inflammation in the pathogenesis, along with complementary factors such as high blood pressure, endothelial dysfunction, and dyslipidemia [42]. Atherosclerotic cardiovascular disease can cause heart attack, angina pectoris, stroke, and transient ischemic attacks as well as aortic aneurysm. 

The development and progression of atherosclerosis is a significant factor contributing to the pathogenesis of cardiovascular diseases. ROS are one of the primary promotors of the progression of atherosclerotic plaque, and this process can be categorized as vascular aging [43]. Moreover, mitochondrial dysfunction, activation of enzymes producing nitrogen types, and a decline in the activity of antioxidant systems are among the causes of increased oxidative stress associated with vascular aging [29,44]. 

The pathogenesis of atherosclerosis is characterized by lipid metabolic disorders in addition to oxidative stress [37]. There are several hypotheses that explain the onset of atherosclerosis, including reaction to injury, oxidative modification, and an inflammatory pattern [45]. One of the first steps encountered in atherosclerosis is the oxidation of low-density lipoprotein (LDL) [43]. The CD36 and SRA-1 receptors expressed by macrophages are able to recognize the modified/oxidized lipoprotein that is present in the subintimal opening of an artery. Macrophages rapidly remove oxidized LDLs. Upon the phagocytic uptake of oxidized LDL particles, the formation of macrophage-derived foam cells occurs. These cells are one of the most readily recognizable cells found in fatty streaks, which are the primary lesions for atherosclerosis. Fatty streak formation, in which inflammatory signaling pathways are additionally involved, is characterized by a substantial accumulation of lipids both within the cells and extracellularly [46].

Carotenoids can prevent the development of atherosclerosis by directly inhibiting the oxidation of LDL as well as prevent oxidative injury to vascular cells and preserve vascular function [47,48]. 

Lycopene and lutein-like carotenoids possess critical benefits in this regard. High amounts of circulating carotenoids have been shown to have a significant influence on lowering systolic blood pressure as well as LDL cholesterol. Lycopene, an oil-soluble carotenoid found in vegetables and fruits, particularly tomatoes, shrimp, red carrots, and olives [45], expresses a high antioxidant potential that leads to a decrease in LDL and increases endothelial function. A recent meta-analysis found that individuals consuming lycopene in their diet demonstrated a 17% reduction in the risk of cardiovascular disease [33]. Vascular nitric oxide (NO) impairment can also be the cause of ROS-related aging and increased atherosclerosis. Unlike the damaged structure, the normal vascular structure is characterized by significant levels of NO [43]. Lycopene can reduce vascular degeneration due to its antioxidant capabilities that increase the vascular bioavailability of lutein and astaxanthin. 

Lutein is 15 times more potent than lycopene and 10 times stronger than β-carotene [46]. It is the most common carotenoid that creates the bright color pigment in spinach-like and dark-green leafy vegetables; egg yolk is also a highly bioavailable source of lutein. Since it scavenges ROS, it is effective in preventing lipid peroxidation [37]. 

The intake of astaxanthin through the diet has also been shown to delay LDL oxidation [29]. One of the antioxidant effects of astaxanthin is a reduced production of superoxide anion radicals leading to lower oxidative stress and higher bioavailability of NO [29,43]. 

Cardiovascular health is significantly influenced by the individual’s diet. In this context, carotenoids might co-contribute to the observation that Mediterranean countries have reduced rates of cardiovascular disease mortality [42,47] since the Mediterranean diet is high in dietary carotenoids from fresh vegetables and fruits.

### 5.2. Skin Health and Carotenoids

Skin is not only the largest but also the most exposed organ of the human body. Skin aging is influenced by both internal and external factors and, hence, genetic as well as environmental factors play a decisive role. Alterations can affect the entire skin or just certain parts and depend on time, hormone status, exposure to UV radiation, cigarette smoke, air pollution, nutritional factors, temperature, stress, and lack of sleep [48]. In this regard, it is important to maintain the skin’s complex defense mechanisms against harmful threats (e.g., microbial infestation, UV radiation), its regeneration efficacy (e.g., wound healing), and function as a sensory organ including temperature regulation [49,50]. Because of internal aging, a decrease in the ability of keratinocytes to reproduce and repair occurs as well as a decrease in the number of epidermal stem cells; both adversely affect skin barrier function [51]. 

Photoaging of skin is environmentally induced, leading to loss of elasticity, pigmentation disorders, dryness, and itchiness [52]. UV rays, the primary cause of photoaging, promote the formation of ROS and adversely impact the skin’s defense and repair mechanisms, causing dermal and epidermal damage and skin aging [53]. Research indicates that carotenoid supplementation may provide protection against UV-induced ROS formation [54].

Lycopene, a carotenoid antioxidant, is recognized as a highly effective singlet oxygen scavenging and peroxyl radical trapping agent [52,55]. Hence, its photoprotective properties were suggested. Several studies indicate that lycopene improves skin photoaging and reduces the intensity of erythema formation [55]. The results of a meta-analysis of nine studies examining the effects of lycopene and tomato on photoaging are contradictory. It was revealed that a diet rich in lycopene reduced skin pigmentation and significantly increased skin thickness and density [56]. However, the discrepancy in the results of the studies may be since tomatoes contain other carotenoids besides lycopene.

Astaxanthin has been shown to improve skin health by inhibiting oxidative damage, and it functions as an anti-photoaging agent [57]. Due to its potency as a coloring agent, it has been discovered that, when taken orally, it can inhibit the synthesis of melanin and decrease hyperpigmentation. According to one of the earliest studies on astaxanthin, it increases skin moisture and can be used to treat dehydrated skin [58]. Other studies supported these findings and confirmed that astaxanthin increases skin hydration and elasticity and significantly reduces dryness [59]. It has been shown that topical and oral administration of astaxanthin led to a reduction in age spots and wrinkles, as well as an enhancement in skin moisture and tissue repair. In studies evaluating the effect of orally administered astaxanthin on UV-induced skin injury, astaxanthin reduced skin moisture loss and rashes [31]. Moreover, astaxanthin downregulates the expression of xanthine oxidase (XO) as well as the reduced form of nicotinamide adenine dinucleotide phosphate oxidase (NOX), which contribute to ROS generation in sunburn insults [60]. Its positive interference can occur through the DNA repair functions. However, a failure to repair DNA causes UV-induced skin damage, and the DNA repair mechanism is also vital in stimulating enzymes that respond to oxidative stress [61]. Hence, it also prevents lipid peroxidation and benefits skin health by preventing sebum lipid peroxidation [62].

The oral administration of lutein has also been shown to increase the antioxidant level, reduce UV-borne erythema, increase skin hydration, and benefit the enhancement of skin elasticity [49,53]. The carotenoid mixture of lutein and zeaxanthin has been observed to lighten the skin and enhance the skin’s appearance [62]. The effect of zeaxanthin on the severity of wrinkles, skin lines, skin color, and brightness has been studied, and a zeaxanthin-based diet and topical treatment has been found to be effective in improving hydration and can be an alternative treatment for wrinkles [50].

Some studies have shown that β-carotene may have protective properties against photodamage [49,52]. The potential of β-carotene to prevent erythema induced by UV rays has also been studied, indicating protection against UV-induced erythema [63]. Conversely to these data, some studies have found that β-carotene has low UV protection and UV-induced erythema reducing activity, irrespective of topical or oral application [64]. There is a concern about the topical application of β-carotene relating to its ability to overcome the stratum cornea barrier to act in deeper skin layers. The availability of β-carotene can be increased by applying drug-loaded nanostructured lipid carriers [65]. In this respect, β-carotene activity following dermal application should still be considered when evaluating its putative protective skin effects. The oral or topical application of Vitamin A itself can contribute to the fight against photoaging and improve sunburn insults [63].

### 5.3. Liver Diseases and Carotenoids

Dietary carotenoid intake is one recommended basic approach to prevent non-alcoholic liver disease (NAFLD) [66]. A study on dietary carotenoid intake revealed that individuals who consumed a high quantity of carotenoids had a low risk of developing NAFLD [67]. There is no surgical or pharmacotherapeutic treatment option available once the disease has developed [66]. Oxidative stress and other pathways that cause NAFLD lead to fat accumulation in hepatocytes. Antioxidants such as carotenoids can help mitigate the severity of NAFLD and can prevent the progression of simple hepatic steatosis to non-alcoholic steatohepatitis (NASH), a state of chronic inflammation of the liver that can lead to progressive damage [68]. Moreover, carotenoids have been shown to possess protective effects against other liver diseases and obesity and on lipid metabolism [66,67,68,69,70,71]. Carotenoids express these effects through several different pathways including a reduction in oxidative stress as well as signaling pathways in the liver with a reduction in pro-inflammatory cytokines secreted by hepatic macrophages [72,73]. 

It has been demonstrated that dietary lycopene has hepatoprotective properties against numerous liver diseases, including alcoholic and non-alcoholic fatty liver disease [70,71]. The protective effect of lycopene is due to its antioxidant properties, resulting in protection against ROS-induced damage.

Some experimental studies have revealed that β-carotene, the most prevalent carotenoid in the liver, has hepatoprotective effects [72,73,74]. It has been shown that the consumption of β-carotene in the diet can reduce the risk of hepatic steatosis and damage caused by free radicals [74]. Campari strain tomatoes, which contain comparatively more β-carotene than other strains, have been shown in a separate study using an obesity model to ameliorate diet-induced hepatosteatosis [72].

Astaxanthin has also been shown to be quite effective at preventing lipid peroxidation and possesses hepatoprotective properties due to its high antioxidant capacity [73]. A recent study found that astaxanthin therapy regulated lipid metabolism disorders and oxidative stress in mice fed a high-fat diet [13]. 

### 5.4. Diabetes Mellitus and Carotenoids

Diabetes mellitus (DM) is a chronic, progressive, and potentially fatal disease characterized by elevated blood sugar [75]. In 2021, 537 million adults (20–79 years old) live with diabetes, according to the International Diabetes Federation (IDF). This number is expected to increase to 643 million by 2030 and 738 million by 2045, with 6.7 million diabetes-related deaths in 2021 [76].

Type-2-DM (T2DM) is the most common type (85–90%) and is commonly diagnosed in people aged 45 years and older [77]. T2DM is caused by age, genetic factors, obesity, and physical inactivity, with increased body fat being the most significant factor [75]. It is characterized by insulin resistance and high glucose levels due to β cell dysfunction in the pancreas where insulin is produced but insulin receptors do not respond or insulin-responsive cells poorly respond to insulin. However, due to numerous severe complications such as neuropathy, retinopathies, and cardiovascular diseases, it diminishes the quality of life and increases the cost of medical care [78]. 

A large number of studies indicate an inverse relationship between carotenoid intake, serum concentrations and T2DM. It has been demonstrated that carotenoid consumption is inversely proportional to insulin resistance [79]. Carotenoids have additionally been demonstrated to be effective in the treatment of diabetic retinopathy and diabetes-related cardiovascular diseases [80].

The gut microbiota were identified as a potential additional factor between the risk of developing DM and the beneficial effects of dietary carotenoids. Studies on supplementation with carotenoids have revealed that these antioxidant compounds contribute to gut immune homeostasis by preventing dysbiosis of the gut microbiota [80,81]. The association between dysbiosis of the intestinal flora and DM has been well established [81]. 

Numerous studies indicate that a high carotenoid intake and serum concentration are inversely related to T2DM [79,82]. There are also studies indicating that individuals with T2DM have lower β-carotene levels [83]. β-carotene regulates lipid metabolism by influencing mature adipocytes, assuring a reduction in fat tissue, and aiding in the regulation of oxidative stress. It has been demonstrated that those deficient in β-carotene have elevated cholesterol levels [84]. Carotenoid concentrations in the epidermis are associated with a low body mass index, insulin resistance index, and triglyceride concentration. The carotenoid with the highest concentration found in the epidermis was β-carotene [85]. It has been discovered that β-carotene supplementation reduces triglyceride levels and insulin resistance [75]. Similarly, another study revealed that those who consumed more vegetable-derived carotenoids had higher serum β-carotene levels and lower insulin resistance [86]. Moreover, protection studies have demonstrated that ß-carotene supplementation defends against T2DM [87].

Due to its powerful antioxidant properties, astaxanthin may be beneficial in the treatment of diabetes as well, according to a number of works [88]. Studies have shown that astaxanthin preserves β cells and protects against oxidative damage caused by diabetes [83]. A three-week astaxanthin supplementation suppressed lipid peroxidation in obese adults and also showed anti-lipid peroxidation activity in rats [84]. It was indicated that astaxanthin improves hyperglycemia [27]. Intraperitoneally administered astaxanthin has been shown to significantly reduce blood sugar [32] and so did daily oral administration of astaxanthin to diabetic rats [38]. Contradictory to these findings, a recent study indicates that oral astaxanthin supplementation has no effect on blood glucose levels in diabetic rats [89].

Low serum lutein levels have been shown to increase the risk of type 2 diabetes in the elderly [90]. Low lutein is linked to elevated glycated hemoglobin (HbA1c) blood levels, which reflect the average blood sugar levels over a period of 3 months. The results of a recent study indicate that the level of lutein in the diet and blood is inversely proportional to the risk of T2DM [82].

Due to its antioxidant properties, lycopene is believed to reduce oxidative stress in patients with T2DM [91]. Researchers have demonstrated that lycopene has anti-diabetic properties [90]. According to studies, individuals who do not have diabetes consume more lycopene [92]. In addition, research indicates that lycopene may improve insulin resistance and glucose metabolism [93]. Recent research indicates that lycopene consumption reduces HbA1c levels [28,91]. The ability to enhance glucose and lipid metabolism in obese animals may reduce the risk of developing diabetes [94]. The lycopene intake of T2DM patients was found to be lower than that of healthy individuals. The same result was observed in diabetic retinopathy patients [91].

### 5.5. Age-Related Macular Degeneration and Carotenoids

Age-related macular degeneration (AMD) is a disease commonly seen in developed countries that occurs due to photoreceptor damage in mature individuals [95]. AMD is the main cause of blindness in individuals over the age of 65 [16]. The prevalence of the disease is increasing, and it is predicted that the number of patients with AMD worldwide will exceed 288 million by 2040 [96]. Macular degeneration progressively leads to a loss of vision and lowers life quality [95]. The first symptoms of AMD are impaired vision, loss of contrast, and sensitivity and it is characterized by a gradual decrease in functional visual acuity in progressive processes [96]. AMD occurs due to many factors, such as age, smoking, and exposure to sunlight [14].

The complex pathophysiology of AMD is not described here in detail. However, there is evidence that AMD-related loss of vision is associated with a progressive degeneration of the retinal pigment epithelium (RPE) cells [97]. They are predisposed to chronic oxidative stress due to their high level of oxygen consumption and exposure to continuous light stimuli. As one aspect, it is suggested that ROS-induced DNA damage in RPE cells can contribute to the development of AMD, but studies also revealed increased ROS production in RPE cells from AMD patients due to the lost ability to increase superoxide dismutase (SOD) expression during oxidative stress [98]. High ROS levels and weakened cellular defense systems result in injury to the photoreceptors and subsequent apoptosis [97].

Carotenoids have been demonstrated to play a crucial role in maintaining macular health and function [7]. Of all carotenoids, only lutein and zeaxanthin possess well-defined mechanisms to cross the blood–retina barrier and accumulate in the retina to form macular pigments [99]. About two decades ago, a membrane-associated xanthophyll-binding protein in the human macula was first described. This specific zeaxanthin-binding protein mediates the uptake, metabolism, and stabilization of zeaxanthin by the retina [100,101].

The optical density of the macular pigment may indicate the condition of the retina [16]. When exposed to light, the macular pigments are responsible for protecting the retina from oxidative stress. By filtering blue light, zeaxanthin and lutein can prevent the formation of singlet oxygen [14]. It has been demonstrated that lutein supplementation over an extended period improves visual acuity [16]. Lutein and zeaxanthin are recommended for the prevention of retinal diseases, improvement in vision, and reduction in the risk of age-related eye diseases such as age-related macular degeneration [14,99]. Just recently, astaxanthin a further member of the xanthophyll family, especially common in marine environments, has been suggested to protect from light-related retinal damage and retinal dysfunction [95]. Since astaxanthin stretches through the cellular bilayer membrane, it can scavenge ROS in both the inner and outer layer, thereby providing protection against oxidative stress. Two years of supplementation with the combination of astaxanthin, lutein, and zeaxanthin improved the visual functions, contrast sensitivity, and visual acuity of patients with AMD.

### 5.6. Diabetic Retinopathy and Carotenoids

Diabetic retinopathy is a significant diabetic complication and one of the leading causes of vision loss in the elderly [97,102]. Under normal conditions, glycemic control, hypertensive dyslipidemia, long-term diabetes, and genetic factors are risk factors for diabetic retinopathy [103]. Due to hyperglycemia, the retina produces ROS, and its structure is negatively impacted. It is believed that astaxanthin can decrease retinal ROS levels [95]. 

Astaxanthin is advised for both the prevention and early treatment of diabetic retinopathy. Short-term treatment with astaxanthin has shown a protective effect on the retina of diabetic rats [104]. Various studies show that macular pigment and serum carotenoid levels are lower in patients with diabetic retinopathy [105,106].

### 5.7. Dry Eye Diseases and Carotenoids

The prevalence of dry eyes (DED) increases with age [107]. According to a study, the prevalence of DED was found to be 22.8% at ≥50 years among individuals and 11.1% at ages ≥ 75 years in women [108]. Oral treatment with astaxanthin in elderly and middle-aged patients acted to alleviate modest to moderate symptoms [26]. Astaxanthin is believed to be a potential treatment for DED [12]. According to the results of a recent study, a combination of lutein ester, zeaxanthin, and extracts of Frank grape, chrysanthemum, and wolf grape reduce the symptoms of eye disorders such as dry eyes, impaired vision, and eye pain [30]. In addition to this study, it has been observed that the combination of astaxanthin with various antioxidants promotes healthy ocular aging and decreases tear ROS levels [95].

### 5.8. Neurodegenerative Diseases and Carotenoids

Some neurodegenerative diseases are neural disorders characterized by the progressive loss of neurons and protein aggregates. Although these diseases are caused by different factors, oxidative stress is an interesting common factor [109]. Neurons and myelin sheaths are harmed by the accumulation of ROS, oxidized proteins, lipids, and nucleic acids because of chronic oxidative stress, which causes neuro-inflammation, the principal cause of cognitive aging and dysfunction [110,111].

#### 5.8.1. Alzheimer’s Disease

Alzheimer’s disease (AD) is the most prevalent neurodegenerative disease associated with aging, characterized by a progressive decline in cognitive function [112]. The majority of neurodegenerative diseases, including AD, are associated with telomere shortening, which is caused by a number of factors, most notably oxidative stress [113]. The concentrations of the six major carotenoids (α-carotene, β-carotene, α-cryptoxanthin, lutein, lycopene, and zeaxanthin) in the blood plasma of AD patients are significantly lower than those of control subjects [109,114]. In one of the studies, it has been reported that serum antioxidant defense is impaired and vitamin E isoforms appear to have different biological roles in the pathogenesis of this disease [114]. Another study suggested that maintaining high plasma lutein levels may reduce the risk of AD [115]. In addition to carotenoid levels in the blood, carotenoid consumption has been linked to reduced AD risk and cognitive decline rates [109]. The application of diets rich in carotenoids, i.e., higher antioxidant intake, may protect telomere health by reducing oxidative stress, regulating telomere length, and thereby preventing AD [116].

#### 5.8.2. Parkinson’s Disease

Parkinson’s disease (PD) is a common disorder characterized by low dopamine levels due to the loss of dopaminergic neurons, accompanied by motor symptoms including tremor, bradykinesia, rigidity, and speech difficulties, as well as non-motor symptoms including depression and insomnia [117]. Dopamine metabolism irregularities promote the formation of ROS and cause irreversible damage to dopaminergic neurons [118]. Serum concentrations of α-carotene, β-carotene, and lycopene are significantly decreased in PD patients, and this decrease is associated with impaired motor function [119,120]. 

In mice challenged with 1-methyl-4-phenyl-1,2,3,6-tetrahydropyridine (MPTP), lutein treatment prevented increases in Bax and Caspase induced by MPTP and improved motor function [121]. Astaxanthin, another carotenoid, prevents neuroinflammation and protects against neural apoptosis by regulating mitochondrial proteins [122]. The prevention of neurodegenerative conditions, such as oxidative stress and neuroinflammation, is one of the defining characteristics of antioxidant diets, and their potential as treatment mechanisms is deemed promising [123].

## 6. Methods 

This narrative review conforms to the Scale for the Assessment of Narrative Review Articles (SANRA) guidelines [124]. The PubMed and ScienceDirect databases were searched for articles containing the main keywords “carotenoids” or “carotenoid and aging” and “carotenoid and skin” or “carotenoid and liver” or “carotenoid and cardiovascular disease” or “carotenoid and eye diseases” or “carotenoid and diabetes” or “carotenoid and neurodegenerative disease” published in English, between November 2004 and January 2023. Secondary search engines (e.g., Google Scholar) were also utilized, and 209,279 scientific articles were found. Based on extensive searches of scientific sources, the relevant published reports that contain the main keywords, with a strong focus on more recently published studies, were included in this narrative review. Preclinical, clinical, observational, or case–control, prospective cohort studies, as well as reviews and meta-analyses were investigated. No exclusion criteria based on the participant’s gender or age were applied.

## 7. Conclusions

Oxidative stress can be shown as a common cause of aging and aging-related diseases. Low levels of oxidative stress may be effective in reducing the prevalence of these diseases and preventing disease progression. It has been observed that carotenoids can be effective in preventing aging-related diseases based on the decreasing oxidative stress and the formation of ROS. Serum or dietary intake of carotenoids can be used in the treatment and prevention of these diseases due to their antioxidant, anti-inflammatory, and anti-aging properties. More studies are needed to clearly determine the effects of an appropriate dose of carotenoid supplementation on aging and aging-related diseases.

## Figures and Tables

**Table 1:** Chemical structures and sources of carotenoids.

Carotenoids	Sources	References
astaxanthin 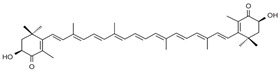	Microalgae (*Haematococcus pluvialis*), pineapples (*Phaffia rhodozyma*), some fungi and bacteria, salmon, krill, peaches, sturgeons, carrots, shells	[12,13]
zeaxanthin 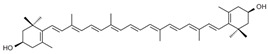	Corn, lavender, spinach, lettuce, broccoli, egg yolk, green leafy vegetables	[14]
β-carotene 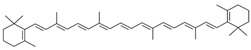	Carrot, orange, sweet potatoes, broccoli, mango	[2,7]
lutein 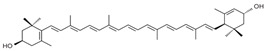	Egg yolk, lettuce, parsley, spinach, basil	[2,15,16,17]
lycopene 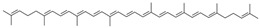	Tomato, rosehip, watermelon, grapefruit, guava, papaya	[18,19,20]

**Table 2:** Studies that investigated the effect of carotenoids on different organisms with aging-related diseases.

Year	Species	Findings on Carotenoid Intake	References
2022	Mice	Astaxanthin significantly reduced HFD-induced fat deposition, obesity, and fatty liver.	[13]
2021	Human	Oral administration of astaxanthin improved symptoms and signs in middle-aged and elderly patients with mild to moderate DED.	[26]
2021	Mice	Astaxanthin improved periodontal destruction and oxidative systemic complications triggered by hyperglycemia in type I diabetes.	[27]
2021	Human	Lycopene intake and peripheral antioxidant level in T2DM patients had a positive correlation while HbA1c and FPG levels were reduced by higher lycopene intake.	[28]
2020	Human	Dietary astaxanthin intake delayed LDL oxidation.	[29]
2020	Human	The combination of lutein ester, zeaxanthin blackcurrant, chrysanthemum, and goji berry extracts improved eye fatigue, dry eye, and macular function.	[30]
2018	Human	Orally taken astaxanthin reduced skin moisture loss and skin roughness.	[31]
2018	Rat	Astaxanthin administered intraperitoneally significantly lowered blood sugar in rats.	[32]
2017	Human	Dietary lycopene intake reduced the risk of CVD by 17%.	[33]
2017	Human	The product rich in lycopene reduced the intensity of erythema formation.	[34]
2016	Rat	Lycopene improved liver fibrosis and reduced abnormal intra- and extrahepatic angiogenesis in rats with cirrhosis. Rats fed lycopene-rich tomato juice for 5 weeks did not develop NAFLD.	[35]
2016	Human	Oral supplementation (isomers of 10 mg of lutein and 2 mg of zeaxanthin) improved skin conditions and brightened the skin.	[36]
2015	Mice	Dietary lutein intake prevented HFD-induced atherosclerosis in ApoE-deficient mice.	[37]
2015	Rat	Astaxanthin originating from shrimp reduced oxidative damage and prevented pathological changes in diabetic rats. Daily administration of astaxanthin in diabetic rats decreased blood sugar.	[38]
2013	Mice	Astaxanthin application (100 mg/kg) inhibited retinal dysfunction in a mouse model of light-induced retinal damage.	[39]
2011	Guinea pig	Lutein prevented cholesterol deposition in the aortic tissue of atherosclerotic guinea pigs.	[40]
2011	Human	Astaxanthin supplementation for 3 weeks suppressed lipid peroxidation in obese adults in Korea.	[41]

ApoE: apolipoprotein E; CVD: cardiovascular disease; DED: dry eye disease; FPG: fasting plasma glucose; HbA1c: glycated hemoglobin; HFD: high fat diet; LDL: low-density lipoprotein; NAFLD: non-alcoholic fatty liver disease; T2DM: type 2 diabetes mellitus.

**Table 3:** Effect of carotenoid types on some aging-related diseases.

Diseases	Lycopene	Lutein	β-Carotene	Astaxanthin	Zeaxanthin
Cardiovascular diseases	Reducing LDL [1]	Prevention of lipid peroxidation [2]	No data	Increase NO bioavailability [3,4]	No data
Neurodegenerative diseases	Healing of motor function [5] Regulation of the length of the telomer [6]	Improving cognitive function [7]	Healing of motor function [5] Regulation of the length of the telomere [6]	Protection against neural apoptosis and preventing neuroinflammation [8]	No data
Diabetes mellitus	Decreasing HbA1c [9]	No data	Decreasing adipose tissue [10] Decreasing triglyceride and insulin resistance [11]	Protecting β cells [12] Anti-lipid peroxidation activity [13] Decreasing blood sugar [14]	No data
Skin diseases	Photoprotection activity [15]	Improving hydration and elasticity [16,17]	Protection against photodamage and skin cancer [16,18]	Suppressing oxidative damage [19] DNA repair [20]	Anti-wrinkle [15]
Liver diseases	ROS extinguishion and antioxidant effect [22]	No data	Hepatoprotective effect [23]	Lipid peroxidation inhibition [24]	No data
Eye diseases	No data	Increasing visual acuity [25]	No data	Decreasing ROS level in retina [26] Improving the symptoms of DED [27]	No data

DED: dry eye disease; LDL: low-density lipoprotein; NO: nitric oxide; ROS: reactive oxygen species.

## References

[1] Bruins M.J., Van Dael P., Eggersdorfer M. (2019). The Role of Nutrients in Reducing the Risk for Noncommunicable Diseases during Aging. Nutrients.

[2] Tan B.L., Norhaizan M.E. (2019). Carotenoids: How Effective Are They to Prevent Age-Related Diseases?. Molecules.

[3] López-Otín C., Blasco M.A., Partridge L., Serrano M., Kroemer G. (2013). The hallmarks of aging. Cell.

[4] Mariani E., Polidori M.C., Cherubini A., Mecocci P. (2005). Oxidative stress in brain aging, neurodegenerative and vascular diseases: An overview. J. Chromatogr. B. Analyt. Technol. Biomed. Life Sci..

[5] Sztretye M., Dienes B., Gönczi M., Czirják T., Csernoch L., Dux L., Szentesi P., Keller-Pintér A. (2019). Astaxanthin: A Potential Mitochondrial-Targeted Antioxidant Treatment in Diseases and with Aging. Oxid. Med. Cell. Longev..

[6] Wei W., Ji S. (2018). Cellular senescence: Molecular mechanisms and pathogenicity. J. Cell. Physiol..

[7] Eggersdorfer M., Wyss A. (2018). Carotenoids in human nutrition and health. Arch. Biochem. Biophys..

[8] Bhatt T., Patel K. (2020). Carotenoids: Potent to Prevent Diseases Review. Nat. Prod. Bioprospect..

[9] Amengual J. (2019). Bioactive Properties of Carotenoids in Human Health. Nutrients.

[10] Metibemu D.S., Ogungbe I.V. (2022). Carotenoids in Drug Discovery and Medicine: Pathways and Molecular Targets Implicated in Human Diseases. Molecules.

[11] Moran N.E., Mohn E.S., Hason N., Erdman J.W., Johnson E.J. (2018). Intrinsic and Extrinsic Factors Impacting Absorption, Metabolism, and Health Effects of Dietary Carotenoids. Intrinsic and Extrinsic Factors Impacting Absorption, Metabolism, and Health Effects of Dietary Carotenoids. Adv. Nutr..

[12] Alugoju P., Krishna Swamy V.K.D., Anthikapalli N.V.A., Tencomnao T. (2022). Health benefits of astaxanthin against age-related diseases of multiple organs: A comprehensive review. Crit. Rev. Food Sci. Nutr..

[13] Li Y., Liu J., Ye B., Cui Y., Geng R., Liu S., Zhang Y., Guo W., Fu S. (2022). Astaxanthin Alleviates Nonalcoholic Fatty Liver Disease by Regulating the Intestinal Flora and Targeting the AMPK/Nrf2 Signal Axis. J. Agric. Food Chem..

[14] Mrowicka M., Mrowicki J., Kucharska E., Majsterek I. (2022). Lutein and Zeaxanthin and Their Roles in Age-Related Macular Degeneration-Neurodegenerative Disease. Nutrients.

[15] Perry A., Rasmussen H., Johnson E.J. (2009). Xanthophyll (lutein, zeaxanthin) content in fruits, vegetables and corn and egg products. J. Food Compos. Analy..

[16] Feng L., Nie K., Jiang H., Fan W. (2019). Effects of lutein supplementation in age-related macular degeneration. PLoS ONE.

[17] Li L.H., Lee J.C., Leung H.H., Lam W.C., Fu Z., Lo A.C.Y. (2020). Lutein Supplementation for Eye Diseases. Nutrients..

[18] Saini R.K., Rengasamy K.R.R., Mahomoodally F.M., Keum Y.S. (2020). Protective effects of lycopene in cancer, cardiovascular, and neurodegenerative diseases: An update on epidemiological and mechanistic perspectives. Pharm. Res..

[19] Crowe-White K.M., Phillips T.A., Ellis A.C. (2019). Lycopene and cognitive function. J. Nutr. Sci..

[20] Mozos I., Stoian D., Caraba A., Malainer C., Horbańczuk J.O., Atanasov A.G. (2018). Lycopene and Vascular Health. Front. Pharm..

[21] Luo J., Mills K., le Cessi S., Noordam R., van Heemst D. (2020). Ageing, age-related diseases and oxidative stress: What to do next?. Ageing Res. Rev..

[22] Jayedi A., Rashidy-Pour A., Parohan M., Zargar M.S., Shab-Bidar S. (2018). Dietary Antioxidants, Circulating Antioxidant Concentrations, Total Antioxidant Capacity, and Risk of All-Cause Mortality: A Systematic Review and Dose-Response Meta-Analysis of Prospective Observational Studies. Adv. Nutr..

[23] Wang D.D., Li Y., Bhupathiraju S.N., Rosner B.A., Sun Q., Giovannucci E.L., Rimm E.B., Manson J.E., Willett W.C., Stampfer M.J. (2021). Fruit and Vegetable Intake and Mortality: Results from 2 Prospective Cohort Studies of US Men and Women and a Meta-Analysis of 26 Cohort Studies. Circulation.

[24] Rao A.V., Rao L.G. (2007). Carotenoids and human health. Pharm. Res..

[25] Therond P. (2006). Dommages créés aux biomolécules (lipides, protéines, ADN) par le stress oxydant [Oxidative stress and damages to biomolecules (lipids, proteins, DNA)]. Ann. Pharm. Fr..

[26] Tian L., Wen Y., Li S., Zhang P., Wang Y., Wang J., Cao K., Du L., Wang N., Jie Y. (2022). Benefits and Safety of Astaxanthin in the Treatment of Mild-to-Moderate Dry Eye Disease. Front. Nutr..

[27] Bhattarai G., So H.S., Kieu T.T.T., Kook S.H., Lee J.C., Jeon Y.M. (2021). Astaxanthin Inhibits Diabetes-Triggered Periodontal Destruction, Ameliorates Oxidative Complications in STZ-Injected Mice, and Recovers Nrf2-Dependent Antioxidant System. Nutrients.

[28] Leh H.E., Sopian M.M., Abu Bakar M.H., Lee L.K. (2021). The role of lycopene for the amelioration of glycaemic status and peripheral antioxidant capacity among the Type II diabetes mellitus patients: A case-control study. Ann. Med..

[29] Pereira C.P.M., Souza A.C.R., Vasconcelos A.R., Prado P.S., Name J.J. (2021). Antioxidant and anti-inflammatory mechanisms of action of astaxanthin in cardiovascular diseases. Int. J. Mol. Med..

[30] Kan J., Wang M., Liu Y., Liu H., Chen L., Zhang X., Huang C., Liu B.Y., Gu Z., Du J. (2020). A novel botanical formula improves eye fatigue and dry eye: A randomized, double-blind, placebo-controlled study. Am. J. Clin. Nutr..

[31] Ito N., Seki S., Ueda F. (2018). The Protective Role of Astaxanthin for UV-Induced Skin Deterioration in Healthy People-A Randomized, Double-Blind, Placebo-Controlled Trial. Nutrients.

[32] Zhang B., Xu D. (2018). Protective effects of astaxanthin on diabetic cardiomyopathy in rats. CyTA–J. Food.

[33] Song B., Liu K., Gao Y., Zhao L., Fang H., Li Y., Pei L., Xu Y. (2017). Lycopene and risk of cardiovascular diseases: A meta-analysis of observational studies. Mol. Nutr. Food Res..

[34] Wood S.M., Mastaloudis A.F., Hester S.N., Gray R., Kern D., Namkoong J., Draelos Z.D. (2017). Protective effects of a novel nutritional and phytonutrient blend on ultraviolet radiation-induced skin damage and inflammatory response through aging defense mechanisms. J. Cosmet. Derm..

[35] Martín-Pozuelo G., González-Barrio R., Barberá G.G., Albalat A., García-Alonso J., Mullen W., Mischak H., Periago M.J. (2016). Tomato Juice Consumption Modifies the Urinary Peptide Profile in Sprague-Dawley Rats with Induced Hepatic Steatosis. Int. J. Mol. Sci..

[36] Juturu V., Bowman J.P., Deshpande J. (2016). Overall skin tone and skin-lightening-improving effects with oral supplementation of lutein and zeaxanthin isomers: A double-blind, placebo-controlled clinical trial. Clin. Cosmet. Investig. Dermatol..

[37] Han H., Cui W., Wang L., Xiong Y., Liu L., Sun X., Hao L. (2015). Lutein prevents high fat diet-induced atherosclerosis in ApoE-deficient mice by inhibiting NADPH oxidase and increasing PPAR expression. Lipids.

[38] Sila A., Ghlissi Z., Kamoun Z., Makni M., Nasri M., Bougatef A., Sahnoun Z. (2015). Astaxanthin from shrimp by-products ameliorates nephropathy in diabetic rats. Eur. J. Nutr..

[39] Otsuka T., Shimazawa M., Nakanishi T., Ohno Y., Inoue Y., Tsuruma K., Ishibashi T., Hara H. (2013). Protective effects of a dietary carotenoid, astaxanthin, against light-induced retinal damage. J. Pharm. Sci..

[40] Kim J.E., Leite J.O., DeOgburn R., Smyth J.A., Clark R.M., Fernandez M.L. (2011). A lutein-enriched diet prevents cholesterol accumulation and decreases oxidized LDL and inflammatory cytokines in the aorta of guinea pigs. J. Nutr..

[41] Choi H.D., Kim J.H., Chang M.J., Kyu-Youn Y., Shin W.G. (2011). Effects of astaxanthin on oxidative stress in overweight and obese adults. Phytother. Res..

[42] Hsieh M.J., Huang C.Y., Kiefer R., Lee S.D., Maurya N., Velmurugan B.K. (2022). Cardiovascular Disease and Possible Ways in Which Lycopene Acts as an Efficient Cardio-Protectant against Different Cardiovascular Risk Factors. Molecules.

[43] Wolak T., Paran E. (2013). Can carotenoids attenuate vascular aging?. Vasc. Pharm..

[44] Vona R., Gambardella L., Cittadini C., Straface E., Pietraforte D. (2019). Biomarkers of Oxidative Stress in Metabolic Syndrome and Associated Diseases. Oxid. Med. Cell. Longev..

[45] Kohlmeier L., Kark J.D., Gomez-Gracia E., Martin B.C., Steck S.E., Kardinaal A.F., Ringstad J., Thamm M., Masaev V., Riemersma R. (1997). Lycopene and myocardial infarction risk in the EURAMIC Study. Am. J. Epidemiol..

[46] Hajizadeh-Sharafabad F., Ghoreishi Z., Maleki V., Tarighat-Esfanjani A. (2019). Mechanistic insights into the effect of lutein on atherosclerosis, vascular dysfunction, and related risk factors: A systematic review of in vivo, ex vivo and in vitro studies. Pharm. Res..

[47] Costa-Rodrigues J., Pinho O., Monteiro P.R.R. (2018). Can lycopene be considered an effective protection against cardiovascular disease?. Food Chem..

[48] Csekes E., Račková L. (2021). Skin Aging, Cellular Senescence and Natural Polyphenols. Int. J. Mol. Sci..

[49] Michalak M., Pierzak M., Kręcisz B., Suliga E. (2021). Bioactive Compounds for Skin Health: A Review. Nutrients.

[50] Schwartz S., Frank E., Gierhart D., Simpson P., Frumento R. (2016). Zeaxanthin-based dietary supplement and topical serum improve hydration and reduce wrinkle count in female subjects. J. Cosmet. Derm..

[51] Gu Y., Han J., Jiang C., Zhang Y. (2020). Biomarkers, oxidative stress and autophagy in skin aging. Ageing Res. Rev..

[52] Meléndez-Martínez A.J., Stinco C.M., Mapelli-Brahm P. (2019). Skin Carotenoids in Public Health and Nutricosmetics: The Emerging Roles and Applications of the UV Radiation-Absorbing Colourless Carotenoids Phytoene and Phytofluene. Nutrients.

[53] Michalak M. (2022). Plant-Derived Antioxidants: Significance in Skin Health and the Ageing Process. Int. J. Mol. Sci..

[54] Darvin M.E., Sterry W., Lademann J., Vergou T. (2011). The Role of Carotenoids in Human Skin. Molecules.

[55] Bavarsad N., Mapar M.A., Safaezadeh M., Latifi S.M. (2021). A double-blind, placebo-controlled randomized trial of skin-lightening cream containing lycopene and wheat bran extract on melasma. J. Cosmet. Derm..

[56] Zhang X., Zhou Q., Qi Y., Chen X., Deng J., Zhang Y., Li R., Fan J. (2023). The effect of tomato and lycopene on clinical characteristics and molecular markers of UV-induced skin deterioration: A systematic review and meta-analysis of intervention trials. Crit. Rev. Food Sci. Nutr..

[57] Singh K.N., Patil S., Barkate H. (2020). Protective effects of astaxanthin on skin: Recent scientific evidence, possible mechanisms, and potential indications. J. Cosmet. Derm..

[58] Galasso C., Corinaldesi C., Sansone C. (2017). Carotenoids from Marine Organisms: Biological Functions and Industrial Applications. Antioxidants.

[59] Tominaga K., Hongo N., Karato M., Yamashita E. (2012). Cosmetic benefits of astaxanthin on humans subjects. Acta Biochem. Pol..

[60] Fang Q., Guo S., Zhou H., Han R., Wu P., Han C. (2017). Astaxanthin protects against early burn-wound progression in rats by attenuating oxidative stress-induced inflammation and mitochondria-related apoptosis. Sci. Rep..

[61] Ko J.C., Chen J.C., Wang T.J., Zheng H.Y., Chen W.C., Chang P.Y., Lin Y.W. (2016). Astaxanthin down-regulates Rad51 expression via inactivation of AKT kinase to enhance mitomycin C-induced cytotoxicity in human non-small cell lung cancer cells. Biochem. Pharm..

[62] Zhou X., Cao Q., Orfila C., Zhao J., Zhang L. (2021). Systematic Review and Meta-Analysis on the Effects of Astaxanthin on Human Skin Ageing. Nutrients.

[63] Stahl W., Sies H. (2012). β-Carotene and other carotenoids in protection from sunlight. Am. J. Clin. Nutr..

[64] Balić A., Mokos M. (2019). Do We Utilize Our Knowledge of the Skin Protective Effects of Carotenoids Enough?. Antioxidants.

[65] Hentschel A., Gramdorf S., Müller R.H., Kurz T. (2008). Beta-carotene-loaded nanostructured lipid carriers. J. Food Sci..

[66] Murillo A.G., DiMarco D.M., Fernandez M.L. (2016). The Potential of Non-Provitamin A Carotenoids for the Prevention and Treatment of Non-Alcoholic Fatty Liver Disease. Biology.

[67] Christensen K., Lawler T., Mares J. (2019). Dietary Carotenoids and Non-Alcoholic Fatty Liver Disease among US Adults, NHANES 2003⁻2014. Nutrients.

[68] Elvira-Torales L.I., Martín-Pozuelo G., González-Barrio R., Navarro-González I., Pallarés F.J., Santaella M., García-Alonso J., Sevilla Á., Periago-Castón M.J. (2019). Ameliorative Effect of Spinach on Non-Alcoholic Fatty Liver Disease Induced in Rats by a High-Fat Diet. Int. J. Mol. Sci..

[69] Jiang W., Guo M.H., Hai X. (2016). Hepatoprotective and antioxidant effects of lycopene on non-alcoholic fatty liver disease in rat. World J. Gastroenterol..

[70] Ibrahim I.M., Althagafy H.S., Abd-Alhameed E.K., Al-Thubiani W.S., Hassanein E.H.M. (2022). Promising hepatoprotective effects of lycopene in different liver diseases. Life Sci..

[71] Elvira-Torales L.I., García-Alonso J., Periago-Castón M.J. (2019). Nutritional Importance of Carotenoids and Their Effect on Liver Health: A Review. Antioxidants.

[72] Yilmaz B., Sahin K., Bilen H., Bahcecioglu I.H., Bilir B., Ashraf S., Halazun K.J., Kucuk O. (2015). Carotenoids and non-alcoholic fatty liver disease. Hepatobiliary Surg. Nutr..

[73] Ni Y., Zhuge F., Nagashimada M., Ota T. (2016). Novel Action of Carotenoids on Non-Alcoholic Fatty Liver Disease: Macrophage Polarization and Liver Homeostasis. Nutrients.

[74] Clugston R.D. (2020). Carotenoids and fatty liver disease: Current knowledge and research gaps. Acta Mol. Cell. Biol. Lipids.

[75] Marcelino G., Machate D.J., Freitas K.C., Hiane P.A., Maldonade I.R., Pott A., Asato M.A., Candido C.J., Guimarães R.C.A. (2020). β-Carotene: Preventive Role for Type 2 Diabetes Mellitus and Obesity: A Review. Molecules.

[76] Atlas I.D. Diabetes around the World in 2021. https://diabetesatlas.org/.

[77] Roglic G. (2023). WHO Global Report on Diabetes: A Summary. https://www.who.int/publications/i/item/9789241565257.

[78] Dowarah J., Singh V.P. (2020). Anti-diabetic drugs recent approaches and advancements. Bioorg. Med. Chem..

[79] Cichoński J., Chrzanowski G. (2022). Microalgae as a Source of Valuable Phenolic Compounds and Carotenoids. Molecules.

[80] Lyu Y., Wu L., Wang F., Shen X., Lin D. (2018). Carotenoid supplementation and retinoic acid in immunoglobulin A regulation of the gut microbiota dysbiosis. Exp. Biol. Med..

[81] Li X., Watanabe K., Kimura I. (2017). Gut Microbiota Dysbiosis Drives and Implies Novel Therapeutic Strategies for Diabetes Mellitus and Related Metabolic Diseases. Front. Immunol..

[82] Jiang Y.W., Sun Z.H., Tong W.W., Yang K., Guo K.Q., Liu G., Pan A. (2021). Dietary Intake and Circulating Concentrations of Carotenoids and Risk of Type 2 Diabetes: A Dose-Response Meta-Analysis of Prospective Observational Studies. Adv. Nutr..

[83] Kanwugu O.N., Glukhareva T.V., Danilova I.G., Kovaleva E.G. (2022). Natural antioxidants in diabetes treatment and management: Prospects of astaxanthin. Crit. Rev. Food Sci. Nutr..

[84] Liu J., Shi W.Q., Cao Y., He L.P., Guan K., Ling W.H., Chen Y.M. (2014). Higher serum carotenoid concentrations associated with a lower prevalence of the metabolic syndrome in middle-aged and elderly Chinese adults. Br. J. Nutr..

[85] Matsumoto M., Suganuma H., Shimizu S., Hayashi H., Sawada K., Tokuda I., Ihara K., Nakaji S. (2020). Skin Carotenoid Level as an Alternative Marker of Serum Total Carotenoid Concentration and Vegetable Intake Correlates with Biomarkers of Circulatory Diseases and Metabolic Syndrome. Nutrients.

[86] Matsumoto M., Waki N., Suganuma H., Takahashi I., Kurauchi S., Sawada K., Tokuda I., Misawa M., Ando M., Itoh K. (2020). Association between Biomarkers of Cardiovascular Diseases and the Blood Concentration of Carotenoids among the General Population without Apparent Illness. Nutrients.

[87] Sluijs I., Beulens J.W., Grobbee D.E., van der Schouw Y.T. (2009). Dietary carotenoid intake is associated with lower prevalence of metabolic syndrome in middle-aged and elderly men. J. Nutr..

[88] Satoh T., Gupta R.C., Ramesh C.G. (2016). Chapter 38—Astaxanthin: Health Benefits and Toxicity. Nutraceuticals.

[89] Chen Q., Tao J., Li G., Zheng D., Tan Y., Li R., Tian L., Li Z., Cheng H., Xie X. (2018). Astaxanthin ameliorates experimental diabetes-induced renal oxidative stress and fibronectin by upregulating connexin43 in glomerular mesangial cells and diabetic mice. Eur. J. Pharm..

[90] Pan F., Cui W., Gao L., Shi X., Yang H., Hu Y., Li M. (2022). Serum lutein is a promising biomarker for type 2 diabetes mellitus and diabetic kidney disease in the elderly. J. Clin. Lab. Anal..

[91] Leh H.E., Lee L.K. (2022). Lycopene: A Potent Antioxidant for the Amelioration of Type II Diabetes Mellitus. Molecules.

[92] Quansah D.Y., Ha K., Jun S., Kim S.A., Shin S., Wie G.A., Joung H. (2017). Associations of Dietary Antioxidants and Risk of Type 2 Diabetes: Data from the 2007–2012 Korea National Health and Nutrition Examination Survey. Molecules.

[93] Zhu N.W., Yin X.L., Lin R., Fan X.L., Chen S.J., Zhu Y.M., Zhao X.Z. (2020). Possible mechanisms of lycopene amelioration of learning and memory impairment in rats with vascular dementia. Neural Regen. Res..

[94] Liu Y., Tian Y., Dai X., Liu T., Zhang Y., Wang S., Shi H., Yin J., Xu T., Zhu R. (2023). Lycopene ameliorates islet function and down-regulates the TLR4/MyD88/NF-κB pathway in diabetic mice and Min6 cells. Food Funct..

[95] Giannaccare G., Pellegrini M., Senni C., Bernabei F., Scorcia V., Cicero A.F.G. (2020). Clinical Applications of Astaxanthin in the Treatment of Ocular Diseases: Emerging Insights. Mar. Drugs.

[96] Dziedziak J., Kasarełło K., Cudnoch-Jędrzejewska A. (2021). Dietary Antioxidants in Age-Related Macular Degeneration and Glaucoma. Antioxidants.

[97] Bungau S., Abdel-Daim M.M., Tit D.M., Ghanem E., Sato S., Maruyama-Inoue M., Yamane S., Kadonosono K. (2019). Health Benefits of Polyphenols and Carotenoids in Age-Related Eye Diseases. Oxid. Med. Cell. Longev..

[98] Yang M., Wang Y. (2022). Recent Advances and the Mechanism of Astaxanthin in Ophthalmological Diseases. J. Ophthalmol..

[99] Bhosale P., Larson A.J., Frederick J.M., Southwick K., Thulin C.D., Bernstein P.S. (2004). Identification and characterization of a Pi isoform of glutathione S-transferase (GSTP1) as a zeaxanthin-binding protein in the macula of the human eye. J. Biol. Chem..

[100] Bhosale P., Li B., Sharifzadeh M., Gellermann W., Frederick J.M., Tsuchida K., Bernstein P.S. (2009). Purification and partial characterization of a lutein-binding protein from human retina. Biochem..

[101] Lin K.Y., Hsih W.H., Lin Y.B., Wen C.Y., Chang T.J. (2021). Update in the epidemiology, risk factors, screening, and treatment of diabetic retinopathy. J. Diabetes Investig..

[102] Fung T.H., Patel B., Wilmot E.G., Amoaku W.M. (2022). Diabetic retinopathy for the non-ophthalmologist. Clin. Med..

[103] Li C., Miao X., Li F., Wang S., Liu Q., Wang Y., Sun J. (2017). Oxidative Stress-Related Mechanisms and Antioxidant Therapy in Diabetic Retinopathy. Oxid. Med. Cell. Longev..

[104] Benlarbi-Ben Khedher M., Hajri K., Dellaa A., Baccouche B., Hammoum I., Boudhrioua-Mihoubi N., Dhifi W., Ben Chaouacha-Chekir R. (2019). Astaxanthin inhibits aldose reductase activity in *Psammomys obesus*, a model of type 2 diabetes and diabetic retinopathy. Food Sci. Nutr..

[105] Lawler T., Korger J., Liu Y., Liu Z., Pak J.W., Barrett N., Blodi B., Domalpally A., Johnson E., Wallace R. (2021). Carotenoids in Age-Related Eye Disease Study Investigators. Serum and Macular Carotenoids in Relation to Retinal Vessel Caliber Fifteen Years Later, in the Second Carotenoids in Age-Related Eye Disease Study. Invest. Ophthalmol. Vis. Sci..

[106] Lem D.W., Gierhart D.L., Davey P.G. (2021). A Systematic Review of Carotenoids in the Management of Diabetic Retinopathy. Nutrients.

[107] Stapleton F., Alves M., Bunya V.Y., Jalbert I., Lekhanont K., Malet F., Na K.S., Schaumberg D., Uchino M., Vehof J. (2017). TFOS DEWS II Epidemiology Report. Ocul. Surf..

[108] Farrand K.F., Fridman M., Stillman I.Ö., Schaumberg D.A. (2017). Prevalence of Diagnosed Dry Eye Disease in the United States Among Adults Aged 18 Years and Older. Am. J. Ophthalmol..

[109] Cho K.S., Shin M., Kim S., Lee S.B. (2018). Recent Advances in Studies on the Therapeutic Potential of Dietary Carotenoids in Neurodegenerative Diseases. Oxid. Med. Cell. Longev..

[110] Mittal K., Katare D.P. (2016). Shared links between type 2 diabetes mellitus and Alzheimer’s disease: A review. Diabetes Metab. Syndr..

[111] Glade M.J. (2010). Oxidative stress and cognitive longevity. Nutrition.

[112] Śliwińska S., Jeziorek M. (2021). The role of nutrition in Alzheimer’s disease. Rocz. Panstw. Zakl. Hig..

[113] Boccardi V., Arosio B., Cari L., Bastiani P., Scamosci M., Casati M., Ferri E., Bertagnoli L., Ciccone S., Rossi P.D. (2020). Beta-carotene, telomerase activity and Alzheimer’s disease in old age subjects. Eur. J. Nutr..

[114] Mullan K., Williams M.A., Cardwell C.R., McGuinness B., Passmore P., Silvestri G., Woodside J.V., McKay G.J. (2017). Serum concentrations of vitamin E and carotenoids are altered in Alzheimer’s disease: A case-control study. Alzheimers Dement..

[115] Feart C., Letenneur L., Helmer C., Samieri C., Schalch W., Etheve S., Delcourt C., Dartigues J.F., Barberger-Gateau P. (2016). Plasma Carotenoids Are Inversely Associated With Dementia Risk in an Elderly French Cohort. J. Gerontol. A Biol. Sci. Med. Sci..

[116] Min K.B., Min J.Y. (2017). Association between leukocyte telomere length and serum carotenoid in US adults. Eur. J. Nutr..

[117] Park H.A., Ellis A.C. (2020). Dietary Antioxidants and Parkinson’s Disease. Antioxidants.

[118] Ying A.F., Khan S., Wu Y., Jin A., Wong A.S.Y., Tan E.K., Yuan J.M., Koh W.P., Tan L.C.S. (2020). Dietary Antioxidants and Risk of Parkinson’s Disease in the Singapore Chinese Health Study. Mov. Disord..

[119] Kim J.H., Hwang J., Shim E., Chung E.J., Jang S.H., Koh S.B. (2017). Association of serum carotenoid, retinol, and tocopherol concentrations with the progression of Parkinson’s Disease. Nutr. Res. Pr..

[120] Yang F., Wolk A., Håkansson N., Pedersen N.L., Wirdefeldt K. (2017). Dietary antioxidants and risk of Parkinson’s disease in two population-based cohorts. Mov. Disord..

[121] Nataraj J., Manivasagam T., Thenmozhi A.J., Essa M.M. (2016). Lutein protects dopaminergic neurons against MPTP-induced apoptotic death and motor dysfunction by ameliorating mitochondrial disruption and oxidative stress. Nutr. Neurosci..

[122] Park H.A., Hayden M.M., Bannerman S., Jansen J., Crowe-White K.M. (2020). Anti-Apoptotic Effects of Carotenoids in Neurodegeneration. Molecules.

[123] Agarwal P., Wang Y., Buchman A.S., Holland T.M., Bennett D.A., Morris M.C. (2022). Dietary antioxidants associated with slower progression of parkinsonian signs in older adults. Nutr. Neurosci..

[124] Baethge C., Goldbeck-Wood S., Mertens S. (2019). SANRA—A scale for the quality assessment of narrative review articles. Res. Integr. Peer Rev..

